# Loneliness at Universities: Determinants of Emotional and Social Loneliness among Students

**DOI:** 10.3390/ijerph15091865

**Published:** 2018-08-29

**Authors:** Katharina Diehl, Charlotte Jansen, Kamila Ishchanova, Jennifer Hilger-Kolb

**Affiliations:** 1 Mannheim Institute of Public Health, Social and Preventive Medicine, Medical Faculty Mannheim, Heidelberg University; Ludolf-Krehl-Str. 7-11; 68167 Mannheim, Germany; Charlotte.Jansen@medma.uni-heidelberg.de (C.J.); kishchanova@gmail.com (K.I.); Jennifer.Hilger-Kolb@medma.uni-heidelberg.de (J.H.-K.); 2 Max-Weber-Institute for Sociology, Heidelberg University; Bergheimer Str. 58, 69115 Heidelberg, Germany

**Keywords:** adolescence, university students, transition, loneliness

## Abstract

The transition from school to university is associated with social, structural, and behavioral changes. These changes may be related to feelings of loneliness, which are in turn related to morbidity. The authors’ aim was to quantify loneliness among students and to identify its determinants and its relation to transition-related variables (e.g., changes in weight, diet, or physical activity since the transition from high school to university). Coming from across Germany, 689 students participated in the Nutrition and Physical Activity in Adolescence (NuPhA) survey (16–29 years; 69.5% female). Associations of loneliness with the above-mentioned aspects were analyzed using descriptive statistics and linear regressions. Altogether, 32.4% felt moderately lonely and 3.2%, severely lonely. Emotional loneliness was more common than social loneliness (severe loneliness: 7.7% vs. 3.2%). Both were positively associated with feelings of depression and anxiety. Being married or in a committed relationship seemed to be protective factors for emotional loneliness. Physical inactivity, an immigrant background, and studying social sciences were related to higher social loneliness. Transition-related variables produced mixed results. In conclusion, this study’s findings indicated that loneliness seemed to be prevalent in university students. The authors identified important starting points for interventions to prevent loneliness. Such interventions may help reduce the disease burden in the students’ future professional life.

## 1. Introduction

In the course of life, there are various social transitions from one stage of life to the next [1,2]. One of the major transitions is from high school to university, which is a crucial event in late adolescence related to structural and social changes that impacts relationships, routines, assumptions, and roles [1,2]. University students, based on their increasing independence and individualization in their new role, start making decisions autonomously [3,4,5].

The beginning of studies often comes with a change of domicile and changes in the relationships with family members [6,7]. While this may provide many opportunities, it can also entail risks: being alone in a new environment (e.g., a new city) and not knowing anyone may lead to loneliness.

Loneliness is defined as a situation in which a person experiences a subjective deficiency of social relationships in a quantitative or qualitative way [8]. According to Weiss [9], there are two distinct types of loneliness: a deficiency of close and intimate relationships leading to emotional loneliness and the lack of a network of social relationships leading to social loneliness. Emotional loneliness arises, for example, after a divorce or death of a partner, whereas social loneliness occurs when somebody is not socially integrated, such as into a group of friends who share common interests. Both types of loneliness have to be examined independently, because the satisfaction for the need of emotional loneliness cannot act as a counterbalance for social loneliness, and vice versa [9].

Loneliness, in general, has various effects on health. Among others, it has been associated with a greater risk for all-cause mortality (e.g., [10]), multi-morbidity (e.g., [11]), depression (e.g., [12]), and suicidal behavior (e.g., [13,14]).

Former studies on loneliness among university students have investigated associations with culture [15,16], gender [12,17,18], social media [19], Internet [20,21,22] and smartphone use [23], attachment [24,25,26], mental distress [27], and academic performance [28].

However, little research has been conducted on health and health-related behaviors in the context of loneliness among university students. A cross-national study found associations between loneliness and subjective health status, sleeping problems, short sleep duration, tobacco use, aggressive behavior, injury, and sexual risk behavior [29]. Hayley et al. [30] also found a link between loneliness and sleep problems. Furthermore, a link between loneliness and illness [31] as well as a link between loneliness and stress reaction [32] have been explored.

Therefore, the authors’ aim was to analyze the prevalence of loneliness among university students, to explore determinants of loneliness, and to analyze the relationship of loneliness with changes in health-related behaviors for the university period compared to that of the high school period. They hypothesized that loneliness was prevalent in university students and that it was related to transition-related variables (e.g., change in body weight, perceived stress of transition). In addition, they hypothesized that emotional loneliness and social loneliness differ in their determinants, since previous research underlines the importance of conceptually separating emotional and social loneliness [33].

Data was used from Germany, where the majority of adolescents of a cohort starts studies (58.2% in 2015). The results were therefore important for a large group within a cohort. In Germany, different types of universities exist: universities (Universität) including universities specializing in technical studies (Technische Universität), education (Pädagogische Hochschule), arts (Kunsthochschule), and music (Musikhochschule), as well as universities of applied sciences (Hochschule). Students of all types of universities were included in the survey.

## 2. Materials and Methods

### 2.1. Study Sample

The analysis was based on data from the cross-sectional Nutrition and Physical Activity in Adolescence (NuPhA) Study, an online survey conducted among university students across Germany between 31 October 2014 and 15 January 2015. The online survey was open to university students from every research discipline enrolled at any university in Germany. No further inclusion or exclusion criteria were defined. However, as the authors’ online questionnaire was provided in the German language, only students able to understand German could fill in the questionnaire. To reach as many students as possible, different recruitment strategies were used, such as distributing flyers, sharing the study in social networks and via mailing lists, as well as introducing the study during university lectures. Students were informed about the study’s aims and data security, and that participation was voluntary and withdrawal from the study was possible at any point in time. By selecting the “agreement button” before the start of the actual online survey, each participant gave informed consent. As an incentive, 40 gift cards (20 worth €25, 20 worth €50) were raffled off among all participating students. The online questionnaire was completed by 689 students. The study was approved by the Medical Ethics Committee of the Medical Faculty Mannheim, Heidelberg University (2013-634N-MA).

### 2.2. Measures

Emotional and Social Loneliness. The authors measured emotional loneliness and social loneliness based on the six-item De Jong Gierveld Loneliness Scale [34]. The six-item scale is a reliable and valid measurement instrument for overall, emotional, and social loneliness that is suitable for large surveys [34,35]. The authors focused on reporting the results of emotional and social loneliness separately, because previous theoretical and empirical research underlined the importance of conceptual separation [9,33]. The scale included three items measuring social loneliness and three items measuring emotional loneliness. It had three negatively formulated items (“I miss having people around”, “I experience a general sense of emptiness”, and “I often feel rejected”) and three positively formulated items (“There are many people I can trust completely”, “There are plenty of people I can rely on when I have problems”, and “There are enough people I feel close to”). The items had four response categories: “strongly agree”, “agree”, “disagree”, and “strongly disagree”. Scale scores were calculated by counting “strongly agree” and “agree” on negatively formulated items and “disagree” and “strongly disagree” on positively formulated items. Each loneliness scale ranged from 0 (not emotionally/not socially lonely) to 3 (emotionally/socially lonely). The general loneliness scale ranged from 0 to 6 (0–1 = not lonely, 2–4 = moderately lonely, 5–6 severely lonely). Cronbach’s alpha was 0.681 for emotional loneliness and 0.694 for social loneliness.

Sociodemographic variables. The authors included the following sociodemographic characteristics in their analyses: sex (male/female), age in years (up to 20/21–22/23–24/25 and older), immigrant background (yes/no), family status (married/committed relationship/single), residence (alone/with a partner/shared flat/dormitory or elsewhere), and available money per month in € (up to 550/551–690/690–885/ 886+).

Study-related variables. The authors included the following study-related characteristics in their analyses: change of domicile for studies (yes/no), study discipline (social sciences/medicine and health care/sport sciences/law/other disciplines), and semester (1–3/4–5/6–9/10+). They assigned psychology, linguistic and cultural sciences, natural sciences, and teacher training to “other disciplines” due to the small group sizes.

Health-related variables. The authors included variables related to health and health-related behaviors to examine if these were associated with social and emotional loneliness. They measured body mass index (BMI) (underweight/normal range/overweight) by recording the self-reported weight and height of participants and assigning them according to the international standards for BMI. Furthermore, three categories were used to describe the individual subjective body image: relatively or too thin/just right/relatively or too heavy. Since a relationship between loneliness and depression had been found in previous studies (e.g., [12]), the Patient Health Questionnaire-4 (PHQ-4) was included, which is a four-item measure of depression and anxiety symptoms ranging from 0 to 12. It consists of the PHQ-2 that measures core criteria for depression and the Generalized Anxiety Disorder-2 GAD-2, which is a two-item measure for anxiety [36]. Both have been shown to be excellent screening tools [36]. This continuous variable was categorized into four levels (none/mild/moderate/severe) according to Kroenke et al. [36].

The authors gathered data about smoking (non-smoker/smoker) and alcohol consumption within the last week (yes/no) to measure health-related risk behaviors. The category “non-smoker” also included former smokers. In addition, they measured self-reported physical activity in hours per week and sorted results into four categories (0–1 h/1–2 h/2–4 h/4 h or more).

Transition-related variables. The authors included variables measuring changes in health-related parameters due to the transition from high school to university. They measured self-reported changes in weight (yes/no/I don’t know), direction of weight change (weight increase/weight decrease), diet (yes/no/I don’t know), and physical activity (more activity/less activity/no difference) since the last year at high school. Furthermore, they assessed perceived stress of transition from high school to university using a scale ranging from 1 to 10 (1 = non-stressful, 10 = very stressful). They split perceived stress into three categories using a tertile split (low/medium/high).

### 2.3. Statistical Analysis

The authors analyzed potential group differences between subgroups of sex, immigrant background, change of domicile, and smoking and alcohol consumption regarding emotional and social loneliness by calculating group means and using the Mann-Whitney-U test due to the non-parametric loneliness scales. For variables with more than two categories, they used the Kruskal-Wallis-H test. Additionally, they performed three multiple linear regression models with prior significant variables for each kind of loneliness to analyze the potential associations between emotional and social loneliness with social demographics and health and health-related behaviors. Significant predictors from the first two regression models were used to create a best fit model as the third step. To include categorical variables in regression, the authors generated dummy variables. To identify group differences for the transition-related variables regarding both kinds of loneliness, they again used the above-mentioned procedures for group comparisons. For this study, *p*-values < 0.05 were considered statistically significant. All statistical analyses were performed using IBM SPSS Statistics 22 (IBM Corporation, Armonk, NY, USA).

## 3. Results

Of the participating students, 69.5% were female (see Table 1). On average, participants were 22.69 years old. More than half of the students were in a relationship (56.3%). Two out of ten students lived alone (19.9%). The majority of students changed domicile for studies (74.2%). Furthermore, the majority was not engaged in health-related risk behaviors, and eight out of ten participating students did at least 1–2 h of physical exercise per week (83.3%).

**Figure 1:**
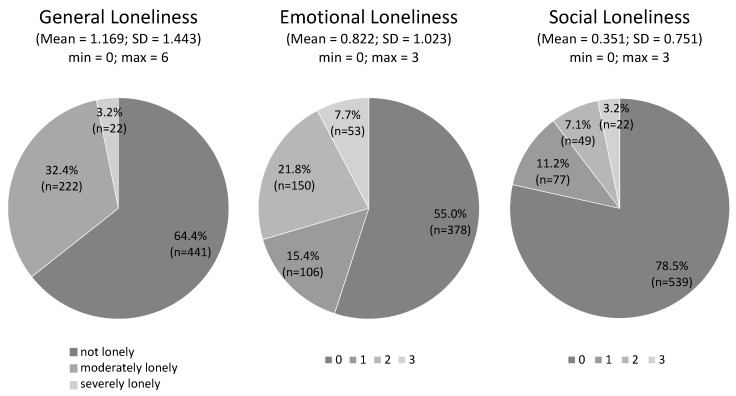
Loneliness in university students in Germany.

According to the general loneliness scale, the majority of students did not feel lonely (64.4%), 32.4% felt moderately lonely, and 3.2% felt severely lonely (see Figure 1). The average loneliness score was 1.169 (SD = 1.443, min = 0, max = 6). More than half of the students felt not emotionally lonely at all (55.0%), 15.4% had a score of 1, 21.8% had a score of 2, and the smallest group had a score of 3 (7.7%) on the emotional loneliness scale. The average emotional loneliness score was 0.882 (SD= 1.023, min = 0, max = 3). Eight out of ten students felt not socially lonely at all (78.5%), 11.2% had a score of 1, 7.1% had a score of 2 and the smallest group had a score of 3 (3.2%) on the social loneliness scale. The average social loneliness score was 0.351 (SD = 0.751, min = 0, max = 3).

The level of emotional loneliness was statistically significant for the different categories of age, family status, residence, available money per month (in €), body image, PHQ-4, and alcohol consumption (see Table 2 and Table 3). The level of emotional loneliness decreased from the youngest to the oldest group of students (*p* = 0.017). Students without a partner had a score of 1.482 for emotional loneliness, whereas students in a relationship or who were married had a much lower score (0.332 and 0.000, respectively; *p* < 0.001). Students living alone were the most emotionally lonely, followed by students living in a dormitory or elsewhere, students living in a shared flat, and students living with a partner (*p* < 0.001). The level of emotional loneliness decreased with the amount of available money per month (*p* = 0.028). Students who perceived themselves as “relatively or too heavy” had the highest emotional loneliness score, followed by “relatively or too thin” and “just right” (*p* = 0.038). Emotional loneliness scores increased with the degree of depression and anxiety measured with the PHQ-4 (*p* < 0.001). Students who had consumed alcohol within the last week were less emotionally lonely than those who had not consumed alcohol (*p* = 0.045).

The level of social loneliness was statistically significant for the different categories of immigrant background, family status, study discipline, PHQ-4, alcohol consumption, and physical activity (see Table 2 and Table 3). Students with an immigrant background were more socially lonely than those with no history of immigration (*p* = 0.038). Students without a partner were the most socially lonely, followed by students in a relationship and those who were married (*p* = 0.018). Students studying social sciences had the highest social loneliness score, whereas law students had the lowest score (*p* = 0.003). Social loneliness scores increased with the degree of depression and anxiety measured with the PHQ-4 (*p* < 0.001). Students who had consumed alcohol within the last week were less socially lonely than those who had not (*p* = 0.035). Students who were physically active 0–1 h per week had the highest social loneliness score, followed by students with 1–2 h and 2 h or more of physical activity (*p* = 0.001).

The linear regression model for emotional loneliness regarding sociodemographics showed an explained variance of 33.2% with significant standardized β coefficients for family status (see Table 4). For health and health-related behaviors, the explained variance was 13.8% with a significant standardized β coefficient for PHQ4. By combining only significant variables in a third regression model, an explained variance of 43.7% was observed. Being married or in a committed relationship was associated with a lower emotional loneliness score, whereas PHQ4 was positively associated with emotional loneliness.

The linear regression model for social loneliness regarding sociodemographics showed an explained variance of 4.9% with significant standardized β coefficients for immigrant background, family status, and field of study (see Table 5). For health and health-related behaviors, the explained variance was 10.7% with significant standardized β coefficients for PHQ4 and physical activity per week. The combination of significant coefficients resulted in an explained variance of 14.3%. Not having an immigrant background was associated with a lower social loneliness score. Furthermore, studying medicine, health care, law, or other disciplines was associated with a lower social loneliness score compared to the social sciences. PHQ4 was positively associated with social loneliness and was its strongest predictor. Being physically active reduced social loneliness.

In addition, group differences were investigated for the transition-related variables regarding emotional and social loneliness, respectively (see Table 6). The level of emotional loneliness differed significantly for the different categories of changes in diet, with the highest score being for students who perceived a change (0.886), followed by those who did not perceive a change (0.727) and those who did not know (0.455; *p* = 0.014). The level of social loneliness was statistically significant for the different categories of changes in weight, with the highest score being for students who perceived a change (0.424), followed by those who did not know (0.413) and those who did not perceive any change (0.240; *p* = 0.006). No differences between groups with emotional and social loneliness scores were found for changes in physical activity and the perceived stress of the transition.

## 4. Discussion

To the best of the authors’ knowledge, they are among the first to analyze loneliness among university students by including health and transition-related aspects. They found that loneliness was prevalent in university students, with 32.4% feeling moderately lonely and 3.2% feeling severely lonely. Emotional loneliness was more common than social loneliness. Both loneliness variables were associated with sociodemographic, health-related, and transition-related determinants.

The authors did not find an association between loneliness and sex. Former studies with different age groups came to mixed results with studies showing higher loneliness among women than men [12,17,18], studies showing higher loneliness in men than women [37,38] and studies that did not find any sex differences [39,40]. In addition, they did not find an association with age, although previous studies had done so. However, while those studies looked at a broader range of age from young adulthood to old age [12,41], the authors focused exclusively on the homogeneous age group of university students.

Emotional loneliness and social loneliness were found to be related but divergent constructs [33]. Besides the association with different determinants as described below, the authors found emotional loneliness being more prevalent than social loneliness. This is congruent with previous research [33]. An explanation for their target group may be that the majority of students was socially well integrated, which led to a lower prevalence in social loneliness. They had friends with shared interests with whom they spent time, e.g., by participating in sports or going to parties. However, these relationships could be rather superficial in that they lacked an emotional component, which led to a higher prevalence of emotional loneliness.

Social loneliness was positively associated with immigrant background, which is in line with the findings of previous studies on loneliness in general [42]. The authors found that emotional loneliness was less frequent in participants in a committed relationship or those being married compared to those not in a relationship. These findings were consistent with the results obtained by Beutel et al. [12] and Nicolaisen and Thorsen [41] who found that loneliness in general was more frequent in participants without a partner. A firm relationship therefore seems to have a protective effect. This is in line with Weiss’ [9] conceptualization.

Furthermore, an association between study discipline and social loneliness was found in this study’s sample. Students studying the social sciences tended to have higher social loneliness scores compared to their fellow students. To the best of the authors’ knowledge, this aspect has not been explored in previous studies and thus needs further research. A study on younger adolescents showed that loneliness may decrease after grade 6, when pupils move from grade schools to secondary schools which tend to have larger and more diverse pupil bodies and pupils have lessons in different classrooms with different compositions [43]. Students in the social sciences are often in small groups and only have contact with other students in minor subjects or during voluntary work at the university. This may lead to a limited number of peers. In addition, there is a higher proportion of self-administered learning and writing in the social sciences. These aspects may perhaps throw light on the explanation of higher social loneliness in the students of social sciences.

Emotional loneliness and social loneliness were both associated with feelings of depression and anxiety. These findings were consistent with the results of previous studies on adults and high school students [12,44]. However, the direction of causality is unclear: Cacioppo et al. [45] found a reciprocal association between depression and loneliness, whereas the results of van Winkel et al. [46] and Cacioppo et al. [47] suggested that loneliness led to depression.

Being physically active was negatively associated with social loneliness among university students. Page and Hammermeister [48] also found that university students without or with infrequent physical activity had higher loneliness scores. This underlines the positive impact of sport activities on social relationships. Intervention studies with older adults revealed that physical activity was effective in reducing loneliness (e.g., [49]). Therefore, physical activity seems to be a protective determinant. The effectiveness of physical activity interventions in university students in reducing loneliness may be worth being investigated in the future.

Regarding changes in health-related behaviors during transition, this study’s results showed the first evidence of an association with loneliness. Changes in diet from high school to university were associated with higher scores in emotional loneliness, whereas changes in weight were associated with lower scores in social loneliness. Since the majority of students reported changes in diet, weight, and physical activity since the start of studies, further investigation of these associations seems important. The authors did not find an association with the change of domicile and the start of studies. For those aspects, students seem to have sufficient coping strategies to deal with their newly structured life.

### Strengths and Limitations

The authors’ study is among the first to analyze loneliness in the age group of university students with a focus on individual, study-related, health-related, and transition-related determinants. Nonetheless, there are some potential limitations. Since all variables were self-reported, the authors cannot rule out a social desirability bias. Transition-related variables required recall of information from memory. Therefore, a recall bias cannot be excluded. Furthermore, even though they recruited university students from across Germany, a potential participation bias cannot be ruled out, which might influence the generalizability of their results. Since they did not recruit students of specific universities based on registries, they were not able to calculate a response rate. However, their approach enabled them to recruit students from all over Germany. Finally, their study does not allow conclusions to be drawn regarding causality due to its cross-sectional design.

## 5. Conclusions

The authors’ findings shed light on the feeling of loneliness among university students. The findings indicated that loneliness was an important topic not only in older ages, but also in adolescence and emerging adulthood. Their results provided the first evidence of an association between loneliness and transition-related changes in health behavior in this age group. Because many adolescents of a cohort begin university studies (e.g., six out of ten in the case of Germany) and because transitions per se represent a major life event, future research is needed to explore this relationship in more detail in order to gain a better understanding of the topic. The authors found physical activity to be a protective factor for social loneliness. Therefore, offering structured university sport programs may help to increase the proportion of physically active students. In addition, loneliness was associated with feelings of depression and anxiety. Introducing and establishing support networks, self-efficacy courses, and contact persons for support and counselling may be valuable. Previous research found that support especially from friends can reduce feelings of loneliness [50]. Therefore, social support groups for beginners may be a helpful tool for discussing potential problems and contacting others, which can both facilitate the students’ start at university [51]. In addition, curricular changes may be helpful, as indicated by Koçak [52], showing that cooperative learning can reduce loneliness. Universities are a perfect setting for conducting interventions to support students in attaining a healthy lifestyle (e.g., by offering sport courses) and also for giving them the opportunity to start their professional career being healthy. Giving support at this stage of life is important in preventing lonely students from “being trapped in loneliness as they age” [53].

## Figures and Tables

**Table 1:** Characteristics of participating university students in Germany.

				Male		Female		
		n	%	n	%	n	%	*p*-Value
Sociodemographics								
Sex	Male	210	30.5					
	Female	479	69.5					
Age	Up to 20	167	24.2	40	19.0	127	26.5	0.081
	21–22	170	24.7	48	22.9	122	25.5	
	23–24	188	27.3	64	30.5	124	25.9	
	25 and older	164	23.8	58	27.6	106	22.1	
Immigrant background	No	593	86.1	178	84.8	415	86.6	0.513
	Yes	96	13.9	32	15.2	64	13.4	
Family status	Married	28	4.1	9	4.3	19	4.0	0.537
	Committed relationship	360	52.2	103	49.0	257	53.7	
	Single	301	43.7	98	46.7	203	42.4	
Residence	Alone	137	19.9	47	22.4	90	18.8	0.700
	With a partner	136	19.7	40	19.0	96	20.0	
	Shared flat	246	35.7	75	35.7	171	35.7	
	Dormitory or elsewhere	170	24.7	48	22.9	122	25.5	
Money per month (in €)	Up to 550	174	25.7	46	22.2	128	27.2	0.045
	551–690	139	20.5	34	16.4	105	22.3	
	691–885	180	26.5	58	28.0	122	25.9	
	886+	185	27.3	69	33.3	116	24.6	
Study-related characteristics								
Change of domicile for studies	No	178	25.8	53	25.2	125	26.1	0.813
	Yes	511	74.2	157	74.8	354	73.9	
Study discipline	Social Sciences	86	12.5	20	9.5	66	13.8	0.018
	Medicine/Health Care	369	53.6	104	49.5	265	55.3	
	Sport Sciences	43	6.3	19	9.0	24	5.0	
	Law	46	6.7	21	10.0	25	5.2	
	Other disciplines	145	21.0	46	21.9	99	20.7	
Semester	1–3	234	34.9	64	31.2	170	36.5	0.361
	4–5	127	18.9	42	20.5	85	18.2	
	6–9	187	27.9	55	26.8	132	28.3	
	10+	123	18.3	44	21.5	79	17.0	

SD = standard deviation; *p*-values are based on chi-squared tests; results were based on the Germany-wide Nutrition and Physical Activity (NuPhA) Study in Adolescence.

**Table 2:** Emotional and social loneliness by sociodemographic and study-related characteristics in university students in Germany.

		Emotional Loneliness			Social Loneliness		
		Mean	SD	*p*-Value	Mean	SD	*p*-Value
Sociodemographics							
Sex	Male	0.708	0.964	0.055	0.367	0.735	0.408
	Female	0.872	1.045		0.344	0.758	
Age	Up to 20	0.970	0.990	0.017	0.353	0.761	0.281
	21–22	0.882	1.068		0.405	0.783	
	23–24	0.734	1.005		0.367	0.773	
	25 and older	0.712	1.017		0.274	0.677	
Immigrant background	No	0.799	1.006	0.178	0.318	0.698	0.038
	Yes	0.969	1.119		0.558	0.997	
Family status	Married	0.000	0.000	<0.001	0.107	0.315	0.018
	Committed relationship	0.332	0.713		0.301	0.712	
Residence	Single	1.482	1.002	<0.001	0.433	0.813	0.375
	Alone	1.073	1.082		0.390	0.809	
	With a partner	0.202	0.610		0.309	0.765	
	Shared flat	0.878	1.015		0.306	0.672	
	Dormitory or elsewhere	1.029	1.057		0.418	0.797	
Money per month (in €)	Up to 550	0.943	1.084	0.028	0.448	0.870	0.464
	551–690	0.921	1.015		0.326	0.685	
	691–885	0.654	0.926		0.333	0.747	
	886+	0.777	1.040		0.299	0.680	
Study-related characteristics							
Change of domicile for studies	No	0.859	1.059	0.634	0.348	0.746	0.849
	Yes	0.810	1.011		0.352	0.753	
Study discipline	Social Sciences	0.954	1.051	0.228	0.659	1.030	0.003
	Medicine/Health Care	0.847	1.050		0.302	0.692	
	Sport Sciences	0.934	1.022		0.419	0.731	
	Law	0.717	0.935		0.152	0.470	
	Other disciplines	0.676	0.957		0.338	0.738	
Semester	1–3	0.944	1.036	0.070	0.389	0.791	0.592
	4–5	0.849	1.059		0.310	0.732	
	6–9	0.738	0.995		0.332	0.701	
	10+	0.721	0.990		0.309	0.737	

*p*-Value based on the Kruskal–Wallis H and Mann–Whitney U tests; emotional and social loneliness assessed based on the six-item De Jong Gierveld Loneliness Scale; scale ranges from 0 to 3; results were based on the Germany-wide Nutrition and Physical Activity (NuPhA) Study in Adolescence.

**Table 3:** Emotional and social loneliness by health and health-related behaviors in university students in Germany.

		Emotional Loneliness			Social Loneliness		
		Mean	SD	*p*-Value	Mean	SD	*p*-Value
Health and health-related behaviors							
BMI	Underweight	0.971	1.087	0.501	0.485	0.712	0.052
	Normal range	0.795	1.003		0.331	0.749	
	Overweight	0.923	1.118		0.407	0.760	
Body image	Relatively or too thin	0.740	0.923	0.038	0.273	0.599	0.083
	Just right	0.717	0.943		0.300	0.709	
	Relatively or too heavy	0.958	1.118		0.430	0.825	
PHQ4	None	0.537	0.830	<0.001	0.232	0.603	<0.001
	Mild	1.005	1.032		0.371	0.762	
	Moderate	1.603	1.256		0.638	0.950	
	Severe	1.654	1.231		1.385	1.169	
Smoking	Non-smoker	0.838	1.023	0.209	0.356	0.751	0.477
	Smoker	0.701	1.027		0.312	0.748	
Alcohol consumption (last week)	No	0.897	1.060	0.045	0.406	0.797	0.035
	Yes	0.737	0.974		0.288	0.689	
Physical activity per week	0–1 h	0.870	1.120	0.517	0.609	0.952	0.001
	1–2 h	0.764	1.001		0.325	0.719	
	2–4 h	0.746	0.960		0.289	0.704	
	4 h or more	0.896	1.040		0.295	0.671	

*p*-Value based on the Kruskal–Wallis H and Mann–Whitney U tests; emotional and social loneliness assessed based on the six-item De Jong Gierveld Loneliness Scale; scale ranges from 0 to 3; PHQ4 = Patient Health Questionnaire-4 (four-item measure of depression and anxiety); results were based on the Germany-wide Nutrition and Physical Activity (NuPhA) Study in Adolescence.

**Table 4:** Regression on emotional loneliness in university students in Germany.

	Model I		Model II		Model III	
	β	*p*-Value	β	*p*-Value	β	*p*-Value
Sociodemographics and emotional loneliness						
Age	0.031	0.367				
Family status						
Married	−0.267	<0.001			−0.272	<0.001
Committed relationship	−0.541	<0.001			−0.544	<0.001
Single	0	Reference			0	Reference
Residence						
Alone	0	Reference				
With a partner	−0.079	0.079				
Shared flat	−0.036	0.396				
Dormitory or elsewhere	−0.034	0.435				
Money per month (in €)	−0.036	0.280				
Health and health-related behaviors and emotional loneliness						
Body image						
Relatively or too thin			−0.007	0.860		
Just right			−0.042	0.284		
Relatively or too heavy			0	Reference		
PHQ4			0.357	<0.001	0.323	<0.001
Alcohol consumption (last week)						
No			0	Reference		
Yes			−0.035	0.338		
R^2^ (n)	0.332 (676)		0.138 (677)		0.437 (678)	

β = standardized β; emotional and social loneliness assessed based on the six-item De Jong Gierveld Loneliness Scale; scale ranges from 0 to 3; PHQ4 = Patient Health Questionnaire-4 (four-item measure of depression and anxiety); results were based on the Germany-wide Nutrition and Physical Activity (NuPhA) Study in Adolescence.

**Table 5:** Regression on social loneliness in university students in Germany.

	Model I		Model II		Model III	
	β	*p*-Value	β	*p*-Value	β	*p*-Value
Sociodemographics and social loneliness						
Immigrant background						
No	−0.099	0.008			−0.072	0.049
Yes	0	Reference			0	Reference
Family status						
Married	−0.073	0.060			−0.071	0.055
Committed relationship	−0.084	0.030			−0.068	0.069
Single	0	Reference			0	Reference
Study discipline						
Social Sciences	0	Reference			0	Reference
Medicine/Health Care	−0.231	<0.001			−0.198	0.001
Sport Sciences	−0.071	0.112			−0.033	0.457
Law	−0.162	<0.001			−0.147	0.001
Other disciplines	−0.169	0.002			−0.147	0.006
Health and health-related behaviors and social loneliness						
PHQ4			0.290	<0.001	0.267	<0.001
Alcohol consumption (last week)						
No			0	Reference		
Yes			−0.034	0.360		
Physical activity per week						
0–1 h			0	Reference	0	Reference
1–2 h			−0.104	0.029	−0.133	0.017
2–4 h			−0.124	0.017	−0.135	0.009
4 h or more			−0.108	0.042	−0.130	0.016
R^2^ (n)	0.049 (687)		0.107 (678)		0.143 (678)	

β = standardized β; emotional and social loneliness assessed based on the six-item De Jong Gierveld Loneliness Scale; scale ranges from 0 to 3; PHQ4 = Patient Health Questionnaire-4 (four-item measure of depression and anxiety); results were based on the Germany-wide Nutrition and Physical Activity (NuPhA) Study in Adolescence.

**Table 6:** Emotional and social loneliness by transition-related variables in university students in Germany.

	Emotional Loneliness			Social Loneliness		
	Mean	SD	*p*-Value	Mean	SD	*p*-Value
Transition-related variables *						
Change in weight			0.720			0.006
Yes	0.8862	1.075		0.424	0.820	
No	0.706	0.938		0.240	0.625	
I don’t know	1.000	1.033		0.413	0.777	
Direction of weight change			0.780			0.210
Weight increase	0.907	1.100		0.479	0.882	
Weight decrease	0.851	1.033		0.351	0.727	
Changes in diet			0.014			0.223
Yes	0.886	1.019		0.374	0.769	
No	0.727	1.027		0.319	0.718	
I don’t know	0.455	0.963		0.181	0.665	
Changes in physical activity			0.153			0.789
More activity	0.888	1.024		0.320	0.678	
Less activity	0.738	1.001		0.387	0.809	
No difference	0.903	1.070		0.323	0.739	
Perceived stress of transition			0.080			0.300
Low	0.706	0.951		0.348	0.774	
Medium	0.832	1.031		0.289	0.634	
High	0.935	1.079		0.425	0.840	

* Changes in health-related parameters due to transition from school to university; SD = standard deviation; *p*-value based on the Kruskal–Wallis H and Mann–Whitney U tests; emotional and social loneliness assessed based on the six-item De Jong Gierveld Loneliness Scale; scale ranges from 0 to 3; results were based on the Germany-wide Nutrition and Physical Activity (NuPhA) Study in Adolescence.
